# [(*Z*)-Ethyl *N*-isopropyl­thio­carbamato-κ*S*](tricyclo­hexyl­phosphine-κ*P*)gold(I)

**DOI:** 10.1107/S1600536810015801

**Published:** 2010-05-08

**Authors:** Primjira P. Tadbuppa, Edward R. T. Tiekink

**Affiliations:** a Department of Chemistry, National University of Singapore, Singapore 117543; b Department of Chemistry, University of Malaya, 50603 Kuala Lumpur, Malaysia

## Abstract

The Au^I^ atom in the title compound, [Au(C_6_H_12_NOS)(C_18_H_33_P)], is coordinated within a *S*,*P*-donor set that defines a slightly distorted linear geometry [S—Au—P angle = 173.44 (5)°], with the distortion due in part to a close intra­molecular Au⋯O contact [3.023 (4) Å]. The N-bound isopropyl group is disordered over two orientations in a 0.618 (15):0.382 (15) ratio.

## Related literature

For the structural systematics and luminescence properties of phosphinegold(I) carbonimidothio­ates, see: Ho *et al.* (2006 ▶); Ho & Tiekink (2007 ▶); Kuan *et al.* (2008 ▶). For the synthesis, see: Hall *et al.* (1993 ▶).
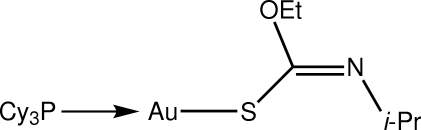


## Experimental

### 

#### Crystal data

[Au(C_6_H_12_NOS)(C_18_H_33_P)]
*M* *_r_* = 623.61
Triclinic, 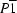

*a* = 9.1226 (6) Å
*b* = 12.3857 (8) Å
*c* = 12.6754 (9) Å
α = 93.475 (1)°
β = 105.380 (2)°
γ = 102.597 (1)°
*V* = 1336.94 (16) Å^3^
*Z* = 2
Mo *K*α radiation
μ = 5.65 mm^−1^
*T* = 223 K
0.23 × 0.15 × 0.08 mm

#### Data collection

Bruker SMART CCD diffractometer
Absorption correction: multi-scan (*SADABS*; Bruker, 2000 ▶) *T*_min_ = 0.607, *T*_max_ = 1
9521 measured reflections
6097 independent reflections
5524 reflections with *I* > 2σ(*I*)
*R*_int_ = 0.023

#### Refinement

*R*[*F* ^2^ > 2σ(*F* ^2^)] = 0.033
*wR*(*F* ^2^) = 0.107
*S* = 1.11
6097 reflections
277 parameters
22 restraints
H-atom parameters constrained
Δρ_max_ = 1.19 e Å^−3^
Δρ_min_ = −1.25 e Å^−3^

### 

Data collection: *SMART* (Bruker, 2000 ▶); cell refinement: *SAINT* (Bruker, 2000 ▶); data reduction: *SHELXTL* (Sheldrick, 2008 ▶); program(s) used to solve structure: *PATTY* in *DIRDIF92* (Beurskens *et al.*, 1992 ▶); program(s) used to refine structure: *SHELXL97* (Sheldrick, 2008 ▶); molecular graphics: *ORTEP-3* (Farrugia, 1997 ▶) and *DIAMOND* (Brandenburg, 2006 ▶); software used to prepare material for publication: *publCIF* (Westrip, 2010 ▶).

## Supplementary Material

Crystal structure: contains datablocks global, I. DOI: 10.1107/S1600536810015801/hb5427sup1.cif

Structure factors: contains datablocks I. DOI: 10.1107/S1600536810015801/hb5427Isup2.hkl

Additional supplementary materials:  crystallographic information; 3D view; checkCIF report

## Figures and Tables

**Fig. 1.**
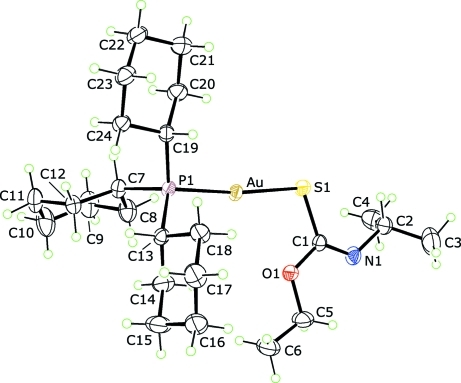
The molecular structure of (I) showing displacement ellipsoids at the 50% probability level. Only the major component of the disordered iso-propyl group is shown for reasons of clarity.

## supplementary crystallographic information

### Comment

The investigation of the title compound, (I), forms a part of systematic structural studies of molecules with the general formula R_3_PAu[SC(OR')═ NR''] for R, R' and R'' = alkyl and aryl (Ho *et al.* 2006; Ho & Tiekink, 2007; Kuan *et al.*, 2008).

In accord with the literature precedents, the gold atom in (I) exists within an SP donor set defined by the phosphine-P and thiolate-S atoms, Table 1 and Fig. 1. The coordination geometry is distorted from the ideal linear [S—Au—P = 173.44 (5) °] owing to the relatively close approach of the O1 atom, 3.023 (4) Å. The carbonimidothioate ligand is functioning as a thiolate as seen in the values of the C1–S1 and C1═N1 bond distances of 1.752 (5) and 1.254 (7) Å, respectively.

### Experimental

Compound (I) was prepared following the standard literature procedure from the reaction of Cy_3_PAuCl and EtOC(═S)N(H)(i-Pr) in the presence of NaOH (Hall *et al.*, 1993). Crystals were obtained by the slow evaporation of a CHCl_3_/hexane (3/1) solution held at room temperature.

### Refinement

The H atoms were geometrically placed (C—H = 0.97-0.99 Å) and refined as riding with *U*_iso_(H) = 1.2-1.5*U*_eq_(C). The maximum and minimum residual electron density peaks of 1.19 and 1.25 e Å^-3^, respectively, were located 0.84 Å and 1.20 Å from the Au atom. High thermal motion was noted in the iso-propyl substituent and two positions were resolved for each of the C atoms. Anisotopic refinement (constrained to be equivalent for paired components of the disorder, and approximately isotropic by the EADP and ISOR commands in SHELXL-97, respectively) and with the C–C distances restrained to 1.52±0.01 Å showed the major component of the disorder had a site occupancy factor = 0.618 (15).

### Crystal data

**Table:**

[Au(C_6_H_12_NOS)(C_18_H_33_P)]	*Z* = 2
*M**_r_* = 623.61	*F*(000) = 628
Triclinic, *P*1	*D*_x_ = 1.549 Mg m^−^^3^
Hall symbol: -P 1	Mo *K*α radiation, λ = 0.71069 Å
*a* = 9.1226 (6) Å	Cell parameters from 4588 reflections
*b* = 12.3857 (8) Å	θ = 2.5–29.5°
*c* = 12.6754 (9) Å	µ = 5.65 mm^−^^1^
α = 93.475 (1)°	*T* = 223 K
β = 105.380 (2)°	Block, colourless
γ = 102.597 (1)°	0.23 × 0.15 × 0.08 mm
*V* = 1336.94 (16) Å^3^	

### Data collection

**Table:**

Bruker SMART CCD diffractometer	6097 independent reflections
Radiation source: fine-focus sealed tube	5524 reflections with *I* > 2σ(*I*)
graphite	*R*_int_ = 0.023
ω scans	θ_max_ = 27.5°, θ_min_ = 1.7°
Absorption correction: multi-scan (*SADABS*; Bruker, 2000)	*h* = −11→10
*T*_min_ = 0.607, *T*_max_ = 1	*k* = −15→16
9521 measured reflections	*l* = −16→15

### Refinement

**Table:**

Refinement on *F*^2^	Primary atom site location: structure-invariant direct methods
Least-squares matrix: full	Secondary atom site location: difference Fourier map
*R*[*F*^2^ > 2σ(*F*^2^)] = 0.033	Hydrogen site location: inferred from neighbouring sites
*wR*(*F*^2^) = 0.107	H-atom parameters constrained
*S* = 1.11	*w* = 1/[σ^2^(*F*_o_^2^) + (0.065*P*)^2^] where *P* = (*F*_o_^2^ + 2*F*_c_^2^)/3
6097 reflections	(Δ/σ)_max_ = 0.001
277 parameters	Δρ_max_ = 1.19 e Å^−^^3^
22 restraints	Δρ_min_ = −1.25 e Å^−^^3^

### Special details

**Table:**

Geometry. All s.u.'s (except the s.u. in the dihedral angle between two l.s. planes) are estimated using the full covariance matrix. The cell s.u.'s are taken into account individually in the estimation of s.u.'s in distances, angles and torsion angles; correlations between s.u.'s in cell parameters are only used when they are defined by crystal symmetry. An approximate (isotropic) treatment of cell s.u.'s is used for estimating s.u.'s involving l.s. planes.
Refinement. Refinement of *F*^2^ against ALL reflections. The weighted *R*-factor wR and goodness of fit *S* are based on *F*^2^, conventional *R*-factors *R* are based on *F*, with *F* set to zero for negative *F*^2^. The threshold expression of *F*^2^ > 2σ(*F*^2^) is used only for calculating *R*-factors(gt) etc. and is not relevant to the choice of reflections for refinement. *R*-factors based on *F*^2^ are statistically about twice as large as those based on *F*, and *R*- factors based on ALL data will be even larger.

### Fractional atomic coordinates and isotropic or equivalent isotropic displacement parameters (Å^2^)

**Table:**

	*x*	*y*	*z*	*U*_iso_*/*U*_eq_	Occ. (<1)
Au	0.26509 (2)	0.057107 (13)	0.194175 (15)	0.03141 (8)	
S1	0.41297 (18)	0.22569 (11)	0.17233 (13)	0.0414 (3)	
P1	0.12363 (15)	−0.11673 (10)	0.19756 (11)	0.0291 (3)	
O1	0.2734 (5)	0.2778 (3)	0.3143 (3)	0.0408 (9)	
N1	0.4305 (6)	0.4290 (4)	0.2737 (4)	0.0406 (10)	
C1	0.3760 (6)	0.3254 (4)	0.2593 (4)	0.0320 (10)	
C2	0.532 (2)	0.4809 (13)	0.213 (2)	0.046 (3)	0.618 (15)
H2	0.5185	0.4296	0.1468	0.056*	0.618 (15)
C3	0.496 (2)	0.5899 (13)	0.1786 (15)	0.088 (4)	0.618 (15)
H3A	0.5467	0.6127	0.1224	0.132*	0.618 (15)
H3B	0.5357	0.6470	0.2420	0.132*	0.618 (15)
H3C	0.3839	0.5796	0.1492	0.132*	0.618 (15)
C4	0.6991 (15)	0.5034 (16)	0.2851 (13)	0.074 (4)	0.618 (15)
H4A	0.7154	0.5590	0.3470	0.111*	0.618 (15)
H4B	0.7697	0.5306	0.2420	0.111*	0.618 (15)
H4C	0.7195	0.4350	0.3121	0.111*	0.618 (15)
C2A	0.560 (4)	0.475 (2)	0.215 (3)	0.046 (3)	0.382 (15)
H2A	0.6035	0.4153	0.1875	0.056*	0.382 (15)
C3A	0.479 (4)	0.534 (2)	0.123 (2)	0.088 (4)	0.382 (15)
H3D	0.5074	0.5162	0.0563	0.132*	0.382 (15)
H3E	0.5118	0.6136	0.1450	0.132*	0.382 (15)
H3F	0.3665	0.5087	0.1086	0.132*	0.382 (15)
C4A	0.682 (3)	0.560 (2)	0.304 (2)	0.074 (4)	0.382 (15)
H4D	0.6450	0.6274	0.3097	0.111*	0.382 (15)
H4E	0.7797	0.5772	0.2849	0.111*	0.382 (15)
H4F	0.6976	0.5300	0.3737	0.111*	0.382 (15)
C5	0.2278 (9)	0.3534 (6)	0.3835 (6)	0.0575 (17)	
H5A	0.1829	0.4074	0.3402	0.069*	
H5B	0.3190	0.3943	0.4434	0.069*	
C6	0.1106 (12)	0.2858 (7)	0.4286 (7)	0.075 (2)	
H6A	0.0209	0.2458	0.3686	0.113*	
H6B	0.0772	0.3341	0.4758	0.113*	
H6C	0.1565	0.2328	0.4713	0.113*	
C7	0.2235 (6)	−0.1951 (4)	0.3003 (5)	0.0380 (11)	
H7	0.2834	−0.2329	0.2618	0.046*	
C8	0.3462 (8)	−0.1216 (6)	0.3977 (5)	0.0538 (16)	
H8A	0.2947	−0.0811	0.4398	0.065*	
H8B	0.4178	−0.0665	0.3707	0.065*	
C9	0.4408 (8)	−0.1896 (6)	0.4740 (6)	0.0632 (19)	
H9A	0.5141	−0.1404	0.5386	0.076*	
H9B	0.5016	−0.2240	0.4348	0.076*	
C10	0.3270 (11)	−0.2814 (7)	0.5114 (7)	0.079 (2)	
H10A	0.3868	−0.3272	0.5568	0.095*	
H10B	0.2741	−0.2462	0.5567	0.095*	
C11	0.2085 (8)	−0.3532 (5)	0.4159 (6)	0.0561 (17)	
H11A	0.1349	−0.4069	0.4426	0.067*	
H11B	0.2609	−0.3954	0.3762	0.067*	
C12	0.1193 (7)	−0.2887 (5)	0.3382 (5)	0.0392 (11)	
H12A	0.0529	−0.2569	0.3743	0.047*	
H12B	0.0502	−0.3398	0.2734	0.047*	
C13	−0.0697 (6)	−0.1169 (4)	0.2183 (4)	0.0324 (10)	
H13	−0.1246	−0.1949	0.2196	0.039*	
C14	−0.0525 (8)	−0.0501 (6)	0.3267 (5)	0.0496 (14)	
H14A	0.0072	0.0264	0.3286	0.059*	
H14B	0.0060	−0.0830	0.3876	0.059*	
C15	−0.2119 (12)	−0.0484 (8)	0.3415 (7)	0.076 (3)	
H15A	−0.2670	−0.1244	0.3464	0.091*	
H15B	−0.1970	−0.0025	0.4110	0.091*	
C16	−0.3115 (10)	−0.0032 (7)	0.2487 (7)	0.068 (2)	
H16A	−0.2630	0.0756	0.2484	0.082*	
H16B	−0.4152	−0.0083	0.2594	0.082*	
C17	−0.3286 (8)	−0.0688 (7)	0.1394 (6)	0.0601 (18)	
H17A	−0.3875	−0.0352	0.0792	0.072*	
H17B	−0.3885	−0.1454	0.1367	0.072*	
C18	−0.1682 (6)	−0.0710 (5)	0.1221 (5)	0.0450 (13)	
H18A	−0.1838	−0.1179	0.0530	0.054*	
H18B	−0.1125	0.0046	0.1165	0.054*	
C19	0.0823 (6)	−0.1990 (4)	0.0622 (4)	0.0337 (10)	
H19	0.0185	−0.1611	0.0078	0.040*	
C20	0.2347 (7)	−0.1946 (5)	0.0286 (6)	0.0462 (14)	
H20A	0.2917	−0.1167	0.0340	0.055*	
H20B	0.3021	−0.2322	0.0795	0.055*	
C21	0.1981 (10)	−0.2509 (6)	−0.0891 (6)	0.0586 (17)	
H21A	0.1396	−0.2088	−0.1406	0.070*	
H21B	0.2963	−0.2505	−0.1070	0.070*	
C22	0.1034 (9)	−0.3689 (5)	−0.1022 (5)	0.0561 (17)	
H22A	0.1661	−0.4128	−0.0561	0.067*	
H22B	0.0769	−0.4018	−0.1792	0.067*	
C23	−0.0453 (8)	−0.3736 (5)	−0.0695 (5)	0.0474 (14)	
H23A	−0.1022	−0.4517	−0.0758	0.057*	
H23B	−0.1125	−0.3360	−0.1205	0.057*	
C24	−0.0122 (6)	−0.3184 (4)	0.0485 (5)	0.0375 (11)	
H24A	−0.1114	−0.3196	0.0652	0.045*	
H24B	0.0460	−0.3602	0.1005	0.045*	

### Atomic displacement parameters (Å^2^)

**Table:**

	*U*^11^	*U*^22^	*U*^33^	*U*^12^	*U*^13^	*U*^23^
Au	0.03193 (12)	0.02433 (12)	0.03862 (12)	0.00659 (8)	0.01104 (8)	0.00564 (7)
S1	0.0477 (8)	0.0273 (6)	0.0540 (8)	0.0038 (6)	0.0272 (7)	0.0036 (6)
P1	0.0273 (6)	0.0248 (6)	0.0360 (6)	0.0074 (5)	0.0089 (5)	0.0056 (5)
O1	0.051 (2)	0.0296 (18)	0.049 (2)	0.0086 (17)	0.0264 (19)	0.0060 (16)
N1	0.047 (3)	0.028 (2)	0.042 (2)	0.002 (2)	0.011 (2)	0.0035 (18)
C1	0.030 (2)	0.030 (2)	0.036 (2)	0.009 (2)	0.008 (2)	0.0079 (19)
C2	0.054 (8)	0.032 (3)	0.052 (3)	0.002 (4)	0.022 (4)	0.004 (3)
C3	0.111 (8)	0.065 (7)	0.088 (8)	0.012 (6)	0.032 (6)	0.033 (6)
C4	0.053 (5)	0.090 (9)	0.081 (6)	0.003 (6)	0.034 (5)	0.008 (7)
C2A	0.054 (8)	0.032 (3)	0.052 (3)	0.002 (4)	0.022 (4)	0.004 (3)
C3A	0.111 (8)	0.065 (7)	0.088 (8)	0.012 (6)	0.032 (6)	0.033 (6)
C4A	0.053 (5)	0.090 (9)	0.081 (6)	0.003 (6)	0.034 (5)	0.008 (7)
C5	0.074 (5)	0.045 (3)	0.058 (4)	0.006 (3)	0.036 (4)	−0.009 (3)
C6	0.108 (7)	0.056 (4)	0.073 (5)	0.005 (4)	0.058 (5)	−0.005 (4)
C7	0.037 (3)	0.033 (3)	0.044 (3)	0.009 (2)	0.010 (2)	0.010 (2)
C8	0.054 (4)	0.051 (4)	0.046 (3)	0.007 (3)	0.001 (3)	0.013 (3)
C9	0.051 (4)	0.064 (4)	0.055 (4)	0.004 (3)	−0.011 (3)	0.015 (3)
C10	0.095 (6)	0.073 (5)	0.057 (4)	0.014 (5)	0.001 (4)	0.033 (4)
C11	0.053 (4)	0.045 (3)	0.072 (4)	0.013 (3)	0.017 (3)	0.029 (3)
C12	0.040 (3)	0.035 (3)	0.041 (3)	0.005 (2)	0.012 (2)	0.010 (2)
C13	0.029 (2)	0.029 (2)	0.040 (3)	0.008 (2)	0.011 (2)	0.006 (2)
C14	0.052 (4)	0.059 (4)	0.042 (3)	0.025 (3)	0.013 (3)	0.002 (3)
C15	0.106 (7)	0.099 (7)	0.055 (4)	0.061 (6)	0.047 (4)	0.022 (4)
C16	0.067 (5)	0.082 (5)	0.086 (5)	0.046 (4)	0.046 (4)	0.035 (4)
C17	0.041 (3)	0.078 (5)	0.073 (5)	0.029 (3)	0.020 (3)	0.031 (4)
C18	0.033 (3)	0.059 (4)	0.051 (3)	0.020 (3)	0.016 (2)	0.019 (3)
C19	0.033 (3)	0.032 (2)	0.038 (3)	0.009 (2)	0.012 (2)	0.008 (2)
C20	0.037 (3)	0.045 (3)	0.063 (4)	0.010 (3)	0.025 (3)	0.004 (3)
C21	0.071 (5)	0.054 (4)	0.064 (4)	0.019 (4)	0.039 (4)	0.006 (3)
C22	0.087 (5)	0.046 (3)	0.044 (3)	0.033 (4)	0.020 (3)	0.003 (3)
C23	0.056 (4)	0.041 (3)	0.043 (3)	0.015 (3)	0.009 (3)	−0.002 (2)
C24	0.034 (3)	0.033 (3)	0.045 (3)	0.007 (2)	0.013 (2)	0.001 (2)

### Geometric parameters (Å, °)

**Table:**

Au—P1	2.2653 (13)	C9—H9B	0.9800
Au—S1	2.3013 (13)	C10—C11	1.476 (11)
S1—C1	1.752 (5)	C10—H10A	0.9800
P1—C7	1.838 (5)	C10—H10B	0.9800
P1—C19	1.845 (5)	C11—C12	1.493 (8)
P1—C13	1.850 (5)	C11—H11A	0.9800
O1—C1	1.365 (6)	C11—H11B	0.9800
O1—C5	1.444 (7)	C12—H12A	0.9800
N1—C1	1.254 (7)	C12—H12B	0.9800
N1—C2A	1.58 (5)	C13—C14	1.512 (8)
N1—C2	1.42 (3)	C13—C18	1.531 (7)
C2—C4	1.511 (9)	C13—H13	0.9900
C2—C3	1.519 (9)	C14—C15	1.519 (10)
C2—H2	0.9900	C14—H14A	0.9800
C3—H3A	0.9700	C14—H14B	0.9800
C3—H3B	0.9700	C15—C16	1.503 (11)
C3—H3C	0.9700	C15—H15A	0.9800
C4—H4A	0.9700	C15—H15B	0.9800
C4—H4B	0.9700	C16—C17	1.516 (12)
C4—H4C	0.9700	C16—H16A	0.9800
C2A—C4A	1.516 (10)	C16—H16B	0.9800
C2A—C3A	1.524 (10)	C17—C18	1.541 (8)
C2A—H2A	0.9900	C17—H17A	0.9800
C3A—H3D	0.9700	C17—H17B	0.9800
C3A—H3E	0.9700	C18—H18A	0.9800
C3A—H3F	0.9700	C18—H18B	0.9800
C4A—H4D	0.9700	C19—C24	1.514 (7)
C4A—H4E	0.9700	C19—C20	1.549 (7)
C4A—H4F	0.9700	C19—H19	0.9900
C5—C6	1.471 (10)	C20—C21	1.526 (10)
C5—H5A	0.9800	C20—H20A	0.9800
C5—H5B	0.9800	C20—H20B	0.9800
C6—H6A	0.9700	C21—C22	1.501 (10)
C6—H6B	0.9700	C21—H21A	0.9800
C6—H6C	0.9700	C21—H21B	0.9800
C7—C8	1.514 (8)	C22—C23	1.511 (9)
C7—C12	1.519 (7)	C22—H22A	0.9800
C7—H7	0.9900	C22—H22B	0.9800
C8—C9	1.538 (9)	C23—C24	1.531 (8)
C8—H8A	0.9800	C23—H23A	0.9800
C8—H8B	0.9800	C23—H23B	0.9800
C9—C10	1.552 (11)	C24—H24A	0.9800
C9—H9A	0.9800	C24—H24B	0.9800
			
P1—Au—S1	173.44 (5)	H11A—C11—H11B	107.8
C1—S1—Au	105.39 (17)	C11—C12—C7	113.5 (5)
C7—P1—C19	106.1 (2)	C11—C12—H12A	108.9
C7—P1—C13	108.4 (2)	C7—C12—H12A	108.9
C19—P1—C13	105.9 (2)	C11—C12—H12B	108.9
C7—P1—Au	114.59 (18)	C7—C12—H12B	108.9
C19—P1—Au	108.69 (16)	H12A—C12—H12B	107.7
C13—P1—Au	112.55 (16)	C14—C13—C18	110.5 (5)
C1—O1—C5	116.3 (4)	C14—C13—P1	111.4 (4)
C1—N1—C2A	116.2 (12)	C18—C13—P1	109.9 (3)
C1—N1—C2	121.2 (9)	C14—C13—H13	108.3
C2A—N1—C2	9.0 (14)	C18—C13—H13	108.3
N1—C1—O1	120.3 (5)	P1—C13—H13	108.3
N1—C1—S1	127.9 (4)	C13—C14—C15	110.9 (6)
O1—C1—S1	111.8 (4)	C13—C14—H14A	109.5
C4—C2—N1	108.7 (15)	C15—C14—H14A	109.5
C4—C2—C3	109.3 (15)	C13—C14—H14B	109.5
N1—C2—C3	111.9 (17)	C15—C14—H14B	109.5
C4—C2—H2	109.0	H14A—C14—H14B	108.0
N1—C2—H2	109.0	C16—C15—C14	112.7 (6)
C3—C2—H2	109.0	C16—C15—H15A	109.0
C4A—C2A—N1	103 (3)	C14—C15—H15A	109.0
C4A—C2A—C3A	110 (2)	C16—C15—H15B	109.0
N1—C2A—C3A	104 (3)	C14—C15—H15B	109.0
C4A—C2A—H2A	113.1	H15A—C15—H15B	107.8
N1—C2A—H2A	113.1	C15—C16—C17	110.0 (6)
C3A—C2A—H2A	113.1	C15—C16—H16A	109.7
C2A—C3A—H3D	109.5	C17—C16—H16A	109.7
C2A—C3A—H3E	109.5	C15—C16—H16B	109.7
H3D—C3A—H3E	109.5	C17—C16—H16B	109.7
C2A—C3A—H3F	109.5	H16A—C16—H16B	108.2
H3D—C3A—H3F	109.5	C16—C17—C18	111.9 (6)
H3E—C3A—H3F	109.5	C16—C17—H17A	109.2
C2A—C4A—H4D	109.5	C18—C17—H17A	109.2
C2A—C4A—H4E	109.5	C16—C17—H17B	109.2
H4D—C4A—H4E	109.5	C18—C17—H17B	109.2
C2A—C4A—H4F	109.5	H17A—C17—H17B	107.9
H4D—C4A—H4F	109.5	C13—C18—C17	110.5 (5)
H4E—C4A—H4F	109.5	C13—C18—H18A	109.6
O1—C5—C6	107.2 (5)	C17—C18—H18A	109.6
O1—C5—H5A	110.3	C13—C18—H18B	109.6
C6—C5—H5A	110.3	C17—C18—H18B	109.6
O1—C5—H5B	110.3	H18A—C18—H18B	108.1
C6—C5—H5B	110.3	C24—C19—C20	110.4 (4)
H5A—C5—H5B	108.5	C24—C19—P1	116.4 (4)
C5—C6—H6A	109.5	C20—C19—P1	111.2 (4)
C5—C6—H6B	109.5	C24—C19—H19	106.0
H6A—C6—H6B	109.5	C20—C19—H19	106.0
C5—C6—H6C	109.5	P1—C19—H19	106.0
H6A—C6—H6C	109.5	C21—C20—C19	111.0 (5)
H6B—C6—H6C	109.5	C21—C20—H20A	109.4
C8—C7—C12	111.1 (5)	C19—C20—H20A	109.4
C8—C7—P1	113.6 (4)	C21—C20—H20B	109.4
C12—C7—P1	116.5 (4)	C19—C20—H20B	109.4
C8—C7—H7	104.8	H20A—C20—H20B	108.0
C12—C7—H7	104.8	C22—C21—C20	111.0 (5)
P1—C7—H7	104.8	C22—C21—H21A	109.4
C7—C8—C9	111.9 (5)	C20—C21—H21A	109.4
C7—C8—H8A	109.2	C22—C21—H21B	109.4
C9—C8—H8A	109.2	C20—C21—H21B	109.4
C7—C8—H8B	109.2	H21A—C21—H21B	108.0
C9—C8—H8B	109.2	C21—C22—C23	111.1 (5)
H8A—C8—H8B	107.9	C21—C22—H22A	109.4
C8—C9—C10	109.4 (6)	C23—C22—H22A	109.4
C8—C9—H9A	109.8	C21—C22—H22B	109.4
C10—C9—H9A	109.8	C23—C22—H22B	109.4
C8—C9—H9B	109.8	H22A—C22—H22B	108.0
C10—C9—H9B	109.8	C22—C23—C24	112.0 (5)
H9A—C9—H9B	108.2	C22—C23—H23A	109.2
C11—C10—C9	111.4 (6)	C24—C23—H23A	109.2
C11—C10—H10A	109.4	C22—C23—H23B	109.2
C9—C10—H10A	109.4	C24—C23—H23B	109.2
C11—C10—H10B	109.4	H23A—C23—H23B	107.9
C9—C10—H10B	109.4	C19—C24—C23	110.4 (5)
H10A—C10—H10B	108.0	C19—C24—H24A	109.6
C10—C11—C12	112.8 (6)	C23—C24—H24A	109.6
C10—C11—H11A	109.0	C19—C24—H24B	109.6
C12—C11—H11A	109.0	C23—C24—H24B	109.6
C10—C11—H11B	109.0	H24A—C24—H24B	108.1
C12—C11—H11B	109.0		
